# Ki-67 immuno-histochemistry index in stage III giant cell tumor of the bone

**DOI:** 10.1186/1756-9966-29-25

**Published:** 2010-03-12

**Authors:** Faisham W Ismail, Aminudin M Shamsudin, Zulmi Wan, Salzihan M Daud, Mutum S Samarendra

**Affiliations:** 1 Musculoskeletal oncology Unit, Orthopedic Department, School of Medical Science, Universiti Sains Malaysia, 16150 Kubang Kerian, Kelantan, Malaysia; 2 Pathology Department, School of Medical Science, Universiti Sains Malaysia, 16150 Kubang Kerian, Kelantan, Malaysia

## Abstract

**Background:**

Giant cell tumor is an infrequent and unpredictable bony lesion. Although numerous attempts have been made to predict the behaviour of GCT, there are no definite biological or histological parameters to determine the prognosis or aggressiveness of this lesion

**Materials and methods:**

We analyzed Ki 67 immuno-histochemistry of 31 consecutive cases staged III giant cell tumor to determine the clinico-pathological correlation. There were 19 male patients compare to 12 females. The mean age was 33.8 years ranged from 18 to 59 years. Five cases presented with local recurrence prior to wide resection and one case had multiple recurrences there after. Six cases had pulmonary metastases. Expression of Ki 67 antigen was evaluated by immuno-histochemical staining techniques using the avidin-biotin perioxidase complex method using an LSAB2 kit (Dako, Glostrup, Denmark). The primary antibody used in this study was Ki-67 (MIB-I clone, dilution 1:25; Dako).

**Results:**

The mean value of Ki-67 index obtained as a percentage of 1000 background cells was 8.15 (ranged 1.00 - 20.0). The median Ki 67 index was 7.5 with standard deviation of 5.12. The Ki 67 index of recurrence tumor was 4.323 compared to 6.05 without recurrence and was not statistically significant (mean difference of 0.865 with p value in independent t test of 0.736). The Ki 67 index was also not statistically significant in the presence of pulmonary metastases with the mean value of metastases group of 6.681 compared to 2.890 without metastases (mean difference of 1.895 with p value in independent t test of 0.424).

**Conclusion:**

Ki 67 index is not use-full prognostic marker for aggressive type of giant cell tumor of the bone.

## Background

Giant cell tumor (GCT) of the bone is an infrequent and unpredictable bony lesion [1]. Although numerous attempts have been made to predict the behaviour of GCT, there are no definite biological or histological parameters to determine the prognosis or aggressiveness of this lesion [2]. Aggressive lesions (stage III Campanacci) are common in oriental population, and they have been shown to have higher risk of recurrence and pulmonary metastases [3-5].

Ki-67 represents a nuclear protein forming part of DNA replicase complex that provides a simple, rapid and reliable means of evaluating the growth fraction of neoplastic cell populations [6]. Ki-67 was shown to correlate with the biological behaviour and risk of pulmonary metastases in a few reported cases of GCT of the bone [7]. However; there are no reported studies to identify the effectiveness of this marker to correlate with the aggressiveness and prognosis of the disease.

The aim of this study is to identify the effectiveness of Ki-67 as prognostic marker and in predicting the risk of local recurrence and pulmonary metastases for aggressive (stage III) GCT of the bone.

## Methods

Thirty-one consecutive patients with histologically proven giant cell tumor, seen at our institution between January 1999 and December 2006, were included. The clinical and radiological records of all the patients were reviewed. Tissue diagnosis and immuno-histopathological study was obtained in all cases from the surgical specimen. Stage III or aggressive GCT in this study is defined as symptomatic, rapidly growing lesion. Bone scans showed intense activity that often extended beyond the lytic area on radiograph and magnetic resonance imaging showed infiltration of the surrounding soft tissue, which was confirmed histologically by tumor that has breached the cortex and extended into the surrounding soft tissue.

There were 19 males and 12 females patents. The mean age was 33.8 years with range from 18 to 59 years. Eleven GCT were located at the proximal tibia followed by 9, involving distal femur, 4 distal radius, 2 distal ulna and one each at the proximal femur, sacrum, metacarpal, distal tibia and proximal humerus. All cases were stage III based on Campanacci staging system^3^. All cases were treated with wide resection margin. The surgical specimens were evaluated for microscopic extent of tumor at the margins and intramedullary marrow extension, and all were found clear of extension. Five cases presented with local recurrence prior to wide resection and one case had multiple recurrences thereafter. Six cases had pulmonary metastases during follow up, two of which underwent surgical resection and four had chemotherapy.

### Evaluation of Ki-67 immuno-histochemical expression

Expression of Ki-67 antigen was evaluated by immuno-histochemical staining in all representative sections from each patient. Serial sections, 5 μm thick, were cut and immuno-histochemical techniques were carried out using the avidin-biotin perioxidase complex method using an LSAB2 kit (Dako, Glostrup, Denmark). The primary antibody used in this study was Ki-67 (MIB-I clone, dilution 1:25; Dako). Expression of proliferation index marker Ki-67 in the nuclear area of the tumor cells were examined using immuno-histochemistry. The labeled-cell count (Ki-67 proliferation index) was determined in ten high-power fields by two blinded observers. Ki-67 proliferation index was defined as the ratio of labeled cells to total cells.

### Statistical analysis

All data obtained were analysed by using SPSS 12.0.1 software. Statistical analysis between different group were determined using independent t-test and considered statistically significant when the p values were less than 0.05.

## Results

The staining was confined to the nuclei of the stromal cells in all cases. The mean value of Ki-67 index obtained as a percentage of 1000 background cells was 8.15 (range 1.00 - 20.0). The median Ki-67 index was 7.5 with standard deviation of 5.12. The Ki-67 index of recurrent tumor was 4.323 as compared to 6.05 without recurrence and was not statistically significant (mean difference of 0.865 with 0.736 of p value in independent t test). The Ki-67 index was also not statistically significant in those with pulmonary metastases with the mean value of 6.68 with metastatic group as compared to 2.89 of those without metastases (mean difference of 1.895 with 0.424 of p value in independent t test).

In the recurrent tumors with pulmonary metastasis, Ki-67 index was 6.40 when compared with 2.20 in disease free cases. The mean difference was 2.099 with p value of 0.326 and was not statistically significant.

## Discussion

Stage III or aggressive giant cell tumor is defined as symptomatic, rapidly growing lesion that is often associated with spontaneous fracture [2,3]. GCT is an infrequent and unpredictable bony lesion, and in our series it was not only presented with locally aggressive behaviour, but it also had higher incidence of local recurrent and pulmonary metastasis [4,5].

Various proliferation markers had been studied to correlate with the aggressive behaviour of GCT and surgical outcome. These included the expression of Ki 67, proliferating cell nuclear antigen, p 53 tumor suppressor gene, matrix metalloproteinase (MMP)-1/9, parathyroid hormone-like protein (PTH-LP) in the mononuclear histiocytic stromal cell. The observations may suggest that mononuclear histiocytic stromal cells are induced by several cytokines acting in an autocrine or paracrine fashion, which are closely related to the biologic aggressiveness of GCT [8-10]. However, most of the studies compared the overall stage of GCT, which were variable in their clinical behaviour. There was no study to quantify the value of proliferative markers in stage III GCT and correlate statistically with the risk of pulmonary metastases. Our series suggest that the Ki-67 index in aggressive type of GCT varies significantly with range between 1.00 to 20.00.

**Figure 1:**
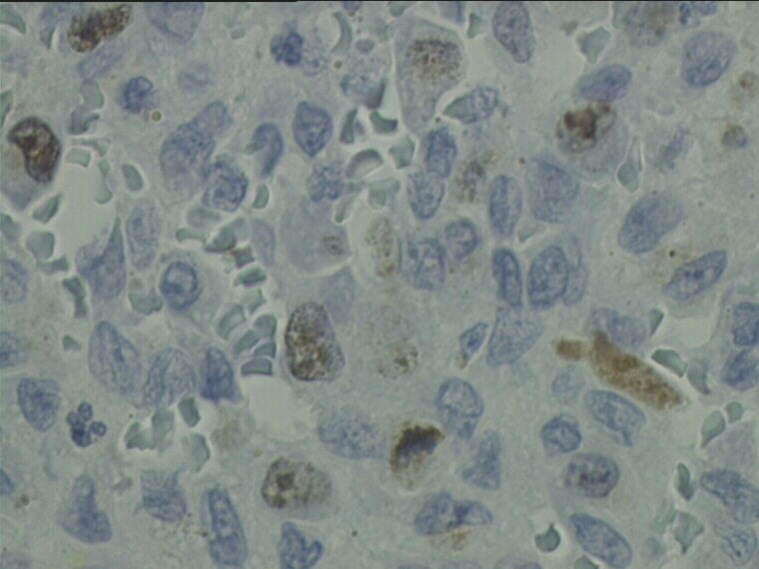
**Photomicrograph shows Ki-67 immuno-histochemical stain (×100)**. Ki-67 labeling in brown is limited to the nuclei of mononuclear stromal cells. The proliferative index was 8.

**Figure 2:**
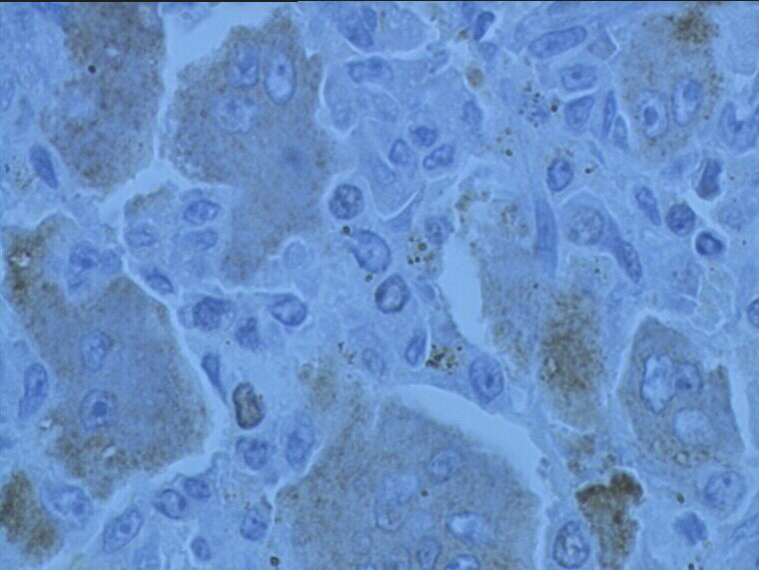
**Photomicrograph shows the Ki-67 of a patient with aggressive GCT of the distal femur and multiple pulmonary metastases**. Despite aggressive clinical behaviour, the Ki-67 index was 2.

The Ki-67 antigen is a human nuclear protein used as a marker for cellular proliferation. The expression is strictly associated with cellular proliferation and is widely used in routine pathological evaluation as a proliferation marker to measure the growth fraction of cells in human tumors. Ki-67 antigen is expressed during the G1, S, G2 and M phases of the cell cycle within the nucleus but is not expressed during the G0 (resting) phase, and thus it is a widely accepted proliferation marker and is useful in predicting the development of human neoplasm [6]. Ki-67 has a short half-life, hence it can be used as a marker for actively proliferating cells. Since it is not expressed during the resting phase of a cell cycle, it functions as a specific indicator of cellular proliferation. Ki-67 antigen immunohistochemistry studies have shown that it is confined to the nuclei of mononuclear cells and there was no labeling of the multinucleated giant cells. This confirms that GCT results from proliferation of mononuclear cells and it is in agreement with our finding in this series that the antigen is confined to the mononuclear stromal cells in all cases. Earlier reports if increase in Ki-67 index in recurrent GCT may indicate that recurrent GCT are more aggressive than the primary tumor [7-10]. In this study the mean value of Ki-67 index of stage III GCT was 8.15. The mean value of Ki-67 index was found to be statistically not significant when tested against the risk of pulmonary metastases and recurrence disease. This was not in agreement with other studies that showed correlation of Ki-67 with aggressiveness of the lesion. (Figure 1) This implies that the proliferative marker Ki-67 may not be useful to predict the risk for tumor recurrent or lung metastases. (Figure 2)

## Conclusion

Ki-67 immuno-pathological marker was not a useful marker to predict the risk of recurrence and pulmonary metastases in aggressive giant cell tumor.

## Competing interests

The authors declare that they have no competing interests.

## Authors' contributions

FWI is the group leader and the work represents his idea in correlation the clinical and basic science of GCT. MSA carried out most of the experimental work, literature review and statistical analysis. MDS and SSM, WZ supervised and evaluated the experimental work, clinical evaluation and also contributed in the discussion and preparation of manuscript.

All authors have read and approved the final manuscript.
